# Decoding pain chronification: mechanisms of the acute-to-chronic transition

**DOI:** 10.3389/fnmol.2025.1596367

**Published:** 2025-06-26

**Authors:** Shunwei Zhang, Youzhi Ning, Yiyi Yang, Guo Mu, Yongkui Yang, Changhe Ren, Changli Liao, Cehua Ou, Yue Zhang

**Affiliations:** ^1^Department of Pain Management, The Affiliated Hospital of Southwest Medical University, Luzhou, China; ^2^Southwest Medical University, Luzhou, China; ^3^Department of Anesthesiology, Zigong Fourth People’s Hospital, Zigong, China; ^4^Department of Pain Management, Hejiang People’s Hospital, Luzhou, China; ^5^Department of Science and Technology, Southwest Medical University, Luzhou, China

**Keywords:** pain chronification, neuroplasticity, neuroinflammation, epigenetic regulation, biomarkers

## Abstract

Pain chronification is a multidimensional and active pathophysiological process, not merely a consequence of prolonged nociception. This review proposes a four-domain mechanistic framework to elucidate the transition from acute to chronic pain. At the molecular-cellular level, persistent neuroinflammation-driven by activated glial cells and pro-inflammatory mediators such as TNF-α and IL-1β-leads to peripheral and central sensitization through enhanced excitability and ion channel dysregulation. In parallel, epigenetic mechanisms such as DNA methylation and histone modifications alter the expression of pain-related genes (e.g., SCN9A, BDNF), establishing a long-term transcriptional predisposition to chronic pain. These changes converge on maladaptive neural plasticity, characterized by aberrant synaptic strengthening, cortical map reorganization, and disrupted functional connectivity, which embed pain into persistent network states. Moreover, psychosocial factors-including catastrophizing, affective distress, and impaired top-down regulation-amplify pain through feedback loops involving the prefrontal cortex, amygdala, and hypothalamic-pituitary-adrenal (HPA) axis. By integrating these four interconnected domains, we highlight critical windows for mechanism-informed, temporally targeted interventions that may interrupt pain chronification and enable a shift toward proactive, personalized pain prevention.

## 1 Introduction

Pain is now widely recognized as a multidimensional experience involving both sensory perception and affective components. It encompasses sensory-discriminative, affective-motivational, and cognitive-evaluative dimensions that together shape the perception of pain. According to the International Association for the Study of Pain (IASP), it may result from actual tissue injury or the perception of threat, and is frequently described within a nociceptive framework (Raja et al., 2020). Based on duration, pain is traditionally classified as acute or chronic. Acute pain usually has a clear underlying cause and resolves within a short timeframe, whereas chronic pain persists for more than 3 months and often lacks a definitive pathological trigger (Treede et al., 2019).

Epidemiological studies reveal that acute pain is common, particularly in postoperative or trauma-related settings. In contrast, chronic pain affects a substantial proportion of the global population and is increasingly recognized as a major public health concern. In the United States, over 20% of adults report living with chronic pain (Yong et al., 2022; Dahlhamer et al., 2018), while in the United Kingdom, estimates suggest that one-third to one-half of the population is affected-approximately 28 million individuals-with higher rates in women and older adults (Fayaz et al., 2016).

Chronic pain significantly impairs quality of life, daily functioning, and work capacity, and is closely associated with psychological comorbidities such as anxiety, depression, sleep disturbances, and reduced social functioning (Nauser et al., 2020; Caputi et al., 2019; Yoshida et al., 2019), leading to an estimated annual economic burden of $635 billion in the United States alone (Pizzo and Clark, 2012). Despite growing interest, current research on pain chronification continues to face three major limitations: fragmented understanding of mechanisms: most studies tend to isolate specific contributors-such as neuroinflammation or cognitive-emotional dysregulation-without offering an integrated model that connects molecular, epigenetic, neural, and psychosocial factors. Translational limitations: although several biomarkers of pain progression have been proposed (e.g., increased IL-6 in cerebrospinal fluid or cortical thinning in the anterior cingulate), their application in guiding therapeutic strategies remains limited. Neglect of temporal dynamics: critical periods for the transition-especially within the first weeks post-injury-remain poorly characterized due to inconsistent study designs and a lack of longitudinal mechanistic analyses.

This review addresses fundamental challenges in pain chronification research through four pivotal advances: first, we establish a novel four-dimensional model integrating molecular-cellular mechanisms (glial-mediated neuroinflammation), epigenetic regulation (DNA methylation of pain-associated genes like SCN9A/BDNF), neuroplastic remodeling (synaptic potentiation and cortical reorganization), and psychosocial amplification (catastrophizing cognition/HPA axis dysregulation), systematically elucidating the dynamic coupling between multi-level biological events and behavioral phenotypes. Crucially, we identify synergistic cross-scale interactions through specific nodal points—exemplified by BDNF methylation simultaneously enhancing spinal cord long-term potentiation while disrupting prefrontal-limbic circuitry to exacerbate negative affect, forming self-reinforcing pathological loops. Building on these insights, we propose phase-specific intervention windows: targeting microglial activation within 1–2 weeks post-injury to block neuroinflammatory cascades, followed by DNA methyltransferase inhibitor administration at 2–4 weeks to reverse maladaptive epigenetic imprints, thereby interrupting pathological progression before adaptive plasticity becomes fixed. This framework extends into four strategic directions: establishing mechanism-guided precision timing for interventions, developing multimodal biomarker assessment systems, creating novel targeted therapies, and integrating cognitive-emotional modulation protocols. Collectively, these advances transcend traditional single-axis research paradigms, providing both theoretical foundations and practical pathways for stratified prevention strategies based on molecular signatures and psychosocial risk profiling.

## 2 Acute and chronic pain: mechanisms and classifications

### 2.1 Acute pain

Acute pain refers to a recent onset of pain that is typically short-lived and results from the activation of nociceptive systems, serving as an alarm and defense mechanism (Raja et al., 2020). The physiological mechanism of acute pain involves a response by peripheral nociceptors to direct mechanical, chemical, or thermal stimuli, usually associated with tissue injury or other factors such as drugs, neurotoxins, or inflammatory states. Acute pain signals are transmitted via classic nociceptive pathways through spinal reflex arcs to the central nervous system (Cao et al., 2024). The types and causes of acute pain generally include: traumatic pain: pain resulting from injuries such as fractures or cuts. Postoperative pain: pain caused by tissue damage and inflammation during surgery. Inflammatory pain: pain triggered by tissue inflammation due to infection, immune responses, or other factors (Kent et al., 2017).

### 2.2 Chronic pain

Chronic pain is defined as a pain state that persists for at least 3 months or longer. It represents a sustained pain experience, where patients continue to perceive pain even in the absence of evident acute injury or stimulation (Treede et al., 2019). Chronic pain is often associated with central sensitization, peripheral sensitization, and neuroplasticity. The classification and common types of chronic pain include: neuropathic pain: pain caused by damage or disease in the nervous system, such as diabetic neuropathy or postherpetic neuralgia. Functional pain: pain without clear pathological damage, as seen in conditions like fibromyalgia or irritable bowel syndrome. Psychogenic pain: pain influenced or exacerbated by psychological factors such as mood disorders or psychological trauma. This distinction between acute and chronic pain, along with their underlying mechanisms, is crucial for understanding the transition process and tailoring effective treatment strategies.

## 3 Mechanisms and research progress during the maladaptive transformation from acute nociception to persistent pain states

Acute pain is typically caused by a specific injury or condition, is short-lived, and serves as a normal physiological response to potential harm, helping to prevent further damage. However, when pain persists beyond 3 months, it may evolve into a multifaceted disorder influenced by complex physiological, psychological, and social factors. The progression from acute nociceptive states to persistent pain syndromes involves multiple critical mechanisms, including sustained inflammatory responses (Schumacher, 2024), structural and functional changes in the nervous system, alterations in neuroplasticity, and the long-term impact of psychological and social factors (Landmark et al., 2024). In the early stages, acute pain, if not effectively managed, may lead to prolonged inflammation and immune responses that result in persistent activation and sensitization of the nervous system (Schumacher, 2024). For instance, ongoing neuroinflammation can amplify pain signaling and facilitate its chronicity. Genetic predisposition plays a key role in pain perception and the development of chronic pain. Specific genetic variants, such as those in SCN9A (encoding the sodium channel Nav1.7) (Faux et al., 2023; Reimann et al., 2010) and COMT (encoding catechol-O-methyltransferase) (Vetterlein et al., 2023), have been associated with pain susceptibility. These genetic variations may alter neuronal excitability and the transmission of pain signals, increasing an individual’s risk of developing chronic pain. Biomarkers such as inflammatory mediators (e.g., cytokines, prostaglandins) and neuroinjury markers [e.g., brain-derived neurotrophic factor (BDNF)] (Sikandar et al., 2018) play pivotal roles in the transition process. These markers reflect the state of the nervous and immune systems and may serve as predictors of pain chronification. Psychological factors are also crucial in this transition. Anxiety and depression, common comorbidities among chronic pain patients, not only influence pain perception but also modulate pain processing through neuroendocrine pathways such as the HPA axis (Wertli et al., 2014; Hannibal and Bishop, 2014). These emotional states can perpetuate pain chronicity by exacerbating pain sensitivity and altering regulatory mechanisms. Chronic psychological stress is another contributing factor. Stress activates the sympathetic nervous system and the HPA axis, elevating levels of stress hormones like cortisol. These hormones can modify the structure and function of the nervous system, increasing the intensity and sensitivity of pain signals (Hannibal and Bishop, 2014; Wyns et al., 2023). Social and environmental factors also play significant roles during the maladaptive transformation from acute nociception to persistent pain states. A lack of social support systems, such as family care and emotional support from friends, increases the risk of pain chronicity. Economic hardship may limit access to timely and effective medical care, while high-pressure work environments impose additional physical and psychological burdens, complicating pain management and recovery (Che et al., 2018; Wilson et al., 2022). On the flip side, strong social support systems may mitigate emotional and physical stress through both psychological comfort and tangible resources, thereby strengthening personal resilience against discomfort. Investigating the interplay of these multifaceted risk elements establishes the groundwork for improved forecasting and interception of acute pain progression into chronic conditions. By pinpointing vulnerable populations and designing personalized strategies that address distinct biological pathways, mental health considerations, and community-level factors, clinicians can formulate targeted and impactful clinical protocols. To systematically delineate the pathophysiological divergence between acute and chronic pain states, Table 1 provides a multidimensional comparison encompassing molecular pathways, epigenetic landscapes, neuroplastic reorganization, and psychosocial determinants. This integrative framework underscores the transition from transient nociceptive signaling to self-sustaining maladaptive circuits.

**Table 1:** Characteristics comparison between acute and chronic pain.

Features	Acute pain	Chronic pain
Molecular & cellular mechanisms	Transient peripheral inflammation (TNF-α, IL-1β) Transient peripheral sensitization (Nav1.7/TRPV1 activation) Brief microglial/astrocytic activation	Persistent neuroinflammation (CXCL13/CX3CL1 signaling amplification) Sustained glial activation (microglia-neuron crosstalk) Ion channel dysfunction (Nav1.7 upregulation, NMDAR hyperactivation)
Genetic & epigenetic regulation	Minimal genetic predisposition Absence of DNA methylation reprogramming Reversible histone modifications	Strong association with SCN9A/COMT polymorphisms DNA methylation abnormalities in DRG (e.g., BDNF silencing) H3K27me3-mediated gene suppression
Neuroplasticity alterations	Short-term synaptic potentiation in dorsal horn (AMPA receptor-mediated) Normal functional brain connectivity	Gray matter reduction in anterior cingulate cortex Enhanced default mode network connectivity (mPFC-NAc circuit) Spinal synaptic remodeling (BDNF-TrkB-NR2B axis)
Psychosocial factors	Transient pain catastrophizing Significant pain relief by social support Acute HPA axis stress response	Anxiety/depression-pain feedback loop Economic stress exacerbates central sensitization Chronic HPA axis dysregulation (cortisol rhythm disruption)

TRPV1, transient receptor potential vanilloid 1; Nav1.8, voltage-gated sodium channel subtype 1.8; CXCL13, C-X-C motif chemokine ligand 13; CatS, cathepsin S; AMPAR, α-amino-3-hydroxy-5-methyl-4-isoxazolepropionic acid receptor; mPFC, medial prefrontal cortex.

### 3.1 Molecular and cellular mechanisms

**Figure 1:**
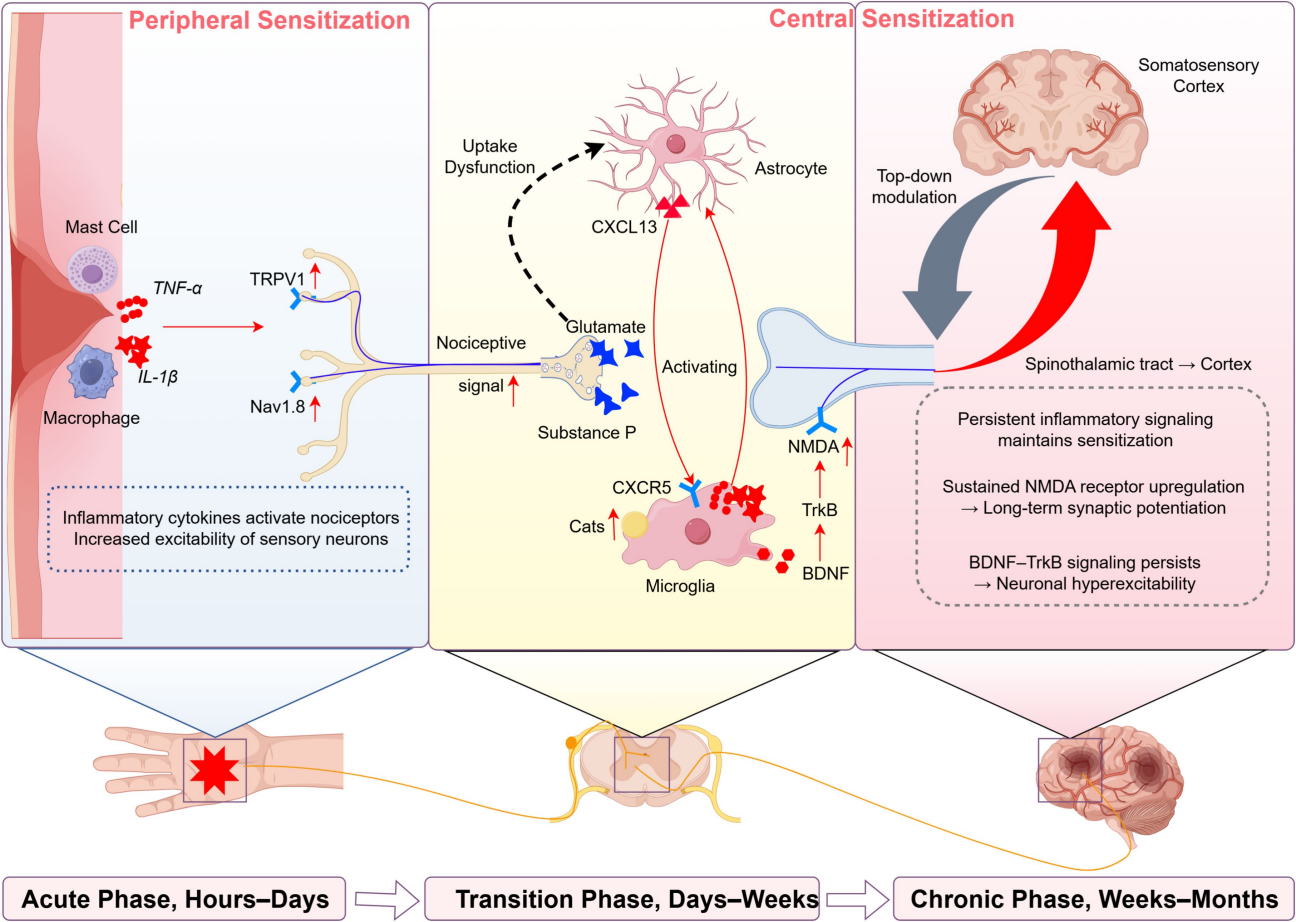
Mechanistic progression of pain chronification: from peripheral sensitization to central maladaptation. This schematic illustrates the sequential biological processes underlying the transition from acute to chronic pain, categorized into three overlapping mechanistic phases-acute (hours–days), transition (days–weeks), and chronic (weeks–months). These phases are defined based on dominant cellular and molecular processes observed in preclinical and translational models, and are not intended to represent fixed clinical thresholds. In the acute phase, tissue injury triggers peripheral immune activation and nociceptor sensitization via pro-inflammatory mediators (e.g., TNF-α, IL-1β), modulating ion channels such as TRPV1 and Nav1.8. The transition phase involves central sensitization, glial activation (e.g., via CXCL13-CXCR5), cathepsin S-driven microglial amplification, and BDNF-TrkB-NMDAR-mediated synaptic plasticity. Chronic pain is maintained by LTP-like changes, maladaptive circuit remodeling, and notably, aberrant top-down modulation from the prefrontal-limbic system, which amplifies nociceptive signals and reinforces affective pain dimensions. Red arrows indicate mechanisms that promote pain chronification, while green arrows (not shown here) represent inhibitory or protective processes in subsequent figures. This temporal-mechanistic framework highlights stage-specific targets for intervention.

The pathophysiological transition from acute to chronic pain involves synergistic interactions across multidimensional mechanisms, where molecular regulatory networks and cellular-level system crosstalk collectively determine critical transformation pathways (Figure 1). At the molecular level, inflammatory mediators such as nerve growth factor (NGF) play a pivotal role by sensitizing nociceptors through dual regulatory strategies. NGF promotes post-translational modifications of nociceptive ion channels such as TRPV1 via phosphorylation, which enhances their gating properties and responsiveness to noxious stimuli (Schumacher, 2024). Simultaneously, NGF activates mitogen-activated protein kinase (MAPK) pathways that upregulate the expression of voltage-gated sodium channels, including Nav1.7 and Nav1.8, thereby contributing to sustained neuronal excitability (Liu et al., 2021; Shi et al., 2018). This bidirectional regulation persistently lowers activation thresholds in sensory neurons, facilitating the maintenance and amplification of pain signals. Crucially, such molecular remodeling establishes the foundation for subsequent cellular processes—a dynamic neuroimmune interplay that orchestrates the progressive development of peripheral and central sensitization through signal amplification cascades (Basbaum et al., 2009). Mechanistically, infiltrating macrophages enhance excitability of peripheral nociceptors via TNF-α and other pro-inflammatory cytokines, whereas microglial activation-induced TLR4/MyD88 signaling triggers long-term potentiation (LTP) in spinal dorsal horn neurons (Xu et al., 2020; Sun et al., 2025). This peripherally-centralized sensitization synergy not only prolongs nociceptive signal transmission but, more significantly, induces persistent maladaptive expression of pain-associated genes through epigenetic modifications. The convergence of these spatiotemporal regulatory events ultimately transforms acute protective nociception into chronic pathological pain states.

#### 3.1.1 Neurons and glial cells: key roles in pain transmission and maintenance

During the transition from acute to chronic pain, persistent nociceptive input not only triggers peripheral neurons to relay pain signals but also induces neuroplastic adaptations within the central nervous system. This transformation shifts neurons from passive signal transmitters to active contributors in pain perpetuation. Within the central nervous system’s (CNS) microenvironment, microglia-the CNS’s intrinsic immune sentinels-undergo morphological activation during systemic infections, propagating inflammatory cascades that critically orchestrate the establishment, exacerbation, and perpetuation of persistent pain syndromes. These glial cells coordinate with circulating monocytes to drive the pathological evolution of pain states from transient protective responses to maladaptive chronic conditions post-neuronal trauma (Li et al., 2020; Peng et al., 2016; Ji et al., 2016).

Astrocytes, another type of glial cell, perform critical functions such as neurotransmitter cycling, blood–brain barrier formation, regulation of extracellular ion concentrations, and modulation of synaptic transmission. Nerve injury induces various astrocytic changes that exacerbate pain (Ji et al., 2016). Additionally, nerve injury upregulates CXCL13 in spinal neurons, which maintains neuropathic pain by activating astrocytes through CXCR5 (Jiang et al., 2016; Zheng et al., 2024). Compared to microglial activation, astrocytic activation is more sustained under chronic pain conditions, indicating their significant contribution to pain chronification.

#### 3.1.2 Neuroimmune crosstalk in the transition from acute to chronic pain

The conscious perception of discomfort frequently correlates with inflammatory signaling—a sophisticated multi-system defense mechanism coordinating neural sensory pathways, immunological surveillance networks, neuroendocrine regulation, and vascular responses to cellular trauma, pathogenic invasion, or noxious agents. This biochemical cascade triggers the targeted migration of specialized leukocyte populations (neutrophil granulocytes, tissue-resident macrophages, adaptive lymphocytes) to compromised anatomical regions. These immunocompetent cells subsequently secrete molecular signaling agents, including protein-based inflammatory regulators, chemotactic guidance molecules, and lipid-derived neuromodulators, which simultaneously calibrate immune activity and lower activation thresholds in peripheral nociceptors, thereby amplifying pain signaling cascades (Ji et al., 2016; Cook et al., 2018; Vanderwall and Milligan, 2019; Ghasemlou et al., 2015).

Nociceptors are activated or sensitized by inflammatory mediators, such as bradykinin, prostaglandins, NGF, and pro-inflammatory cytokines like tumor necrosis factor-alpha (TNF-α), interleukin-1 beta (IL-1β), and pro-inflammatory chemokines (e.g., CCL2, CXCL5). These mediators directly bind and stimulate G-protein-coupled receptors (GPCRs), ionotropic receptors, and tyrosine kinase receptors (Ji et al., 2018). Based on the progression of tissue pathology, inflammation can be categorized into three types: acute inflammation, occurring shortly after injury and lasting for several days; chronic inflammation, persisting for months or even years when acute inflammation fails to resolve; subacute inflammation, representing a transitional phase between acute and chronic inflammation, lasting 2 to 6 weeks (Zotova et al., 2023; Abudukelimu et al., 2018).

The initial inflammatory phase generally elicits nociceptive responses while fulfilling essential protective and homeostatic roles through neutralizing pathogenic threats, activating tissue repair cascades, and reestablishing structural homeostasis. When persisting beyond the subacute phase, this process may evolve into sustained low-grade inflammation. Empirical data from human studies and animal models consistently demonstrate the centrality of immunoregulatory mechanisms in persistent pain pathogenesis. Notably, the lysosomal enzyme CatS emerges as a pivotal biochemical orchestrator within these immune-pain signaling networks (Jiang et al., 2016; Abudukelimu et al., 2018).

#### 3.1.3 Neuroplasticity in pain pathogenesis: synaptic remodeling and glial-mediated adaptations

Neural adaptability-the central nervous system’s capacity for structural and functional reorganization in response to physiological demands-manifests through dynamic modifications in synaptic architecture, neurotransmitter dynamics, and circuit-level information processing. Within nociceptive pathways, this malleability governs the amplification and chronicity of pain states via three principal mechanisms: interneuronal communication remodeling, neurotransmission efficacy modulation, and nociceptive network recalibration. Acute nociception typically induces synaptic potentiation through glutamatergic signaling cascades, whereas persistent pain states correlate with progressive synaptic pruning and circuit desynchronization. These bidirectional modifications fundamentally stem from the CNS’s plastic reorganization capabilities across molecular, cellular, and network hierarchies (Ghasemlou et al., 2015). Recent studies demonstrate that neuroplasticity is a fundamental component of neuropathic pain resulting from nerve injury, occurring both at and distal to the injury site. This maladaptive plasticity is critically regulated by microglia, the CNS’s resident immune cells, which contribute to chronic pain by altering synaptic transmission, modulating neuroinflammatory pathways, and releasing factors such as BDNF, IL-1β, and TNF-α that sensitize nociceptive circuits (Zhou et al., 2019; Inoue and Tsuda, 2018; You et al., 2024). Synaptic plasticity in neurons plays a significant role in the transition from acute to chronic pain. For example, the TRPV1 channel participates in synaptic plasticity, and oxytocin can alleviate neuropathic pain through GABA release in the spinal cord and presynaptic inhibition of TRPV1 (Eliava et al., 2016; Sun et al., 2018).

#### 3.1.4 Changes in ion channels and receptors

Sodium ion channels play a crucial role in pain transmission, particularly voltage-gated sodium channels (VGSCs), which include sodium channels Nav1.7 and Nav1.8. Nav1.7 is primarily expressed in the nervous system, and its dysfunction is associated with various pain disorders (Bennett et al., 2019). For instance, upregulation of Nav1.7 expression and function lead to increased neuronal excitability, which may result in the persistence and intensification of pain (Zeberg et al., 2020). Additionally, mutations in SCN9A, which encodes Nav1.7, enhance channel activity, leading to SCN9A-related neuropathic pain syndrome (SCN9A-NPS) (McDermott et al., 2019; Dib-Hajj et al., 2013; Hisama et al., 1993).

Several drugs and therapeutic approaches have been investigated to target Nav1.7-related pain (Gomez et al., 2023). Non-selective VGSC inhibitors, such as mexiletine, lidocaine, and carbamazepine, have shown efficacy in treating certain Nav1.7-related pain disorders, but their use is limited due to potential effects on VGSC activity in the heart and brain, restricting drug dosage (Yu et al., 2024; Trombetti et al., 2022). Therefore, more selective drugs, such as small molecule inhibitors, peptide toxins, and monoclonal antibodies, are under development. These drugs target specific regions of Nav1.7, such as the voltage sensor or pore region, to provide more precise therapeutic effects while minimizing side effects (Gomez et al., 2023; Trombetti et al., 2022; Safina et al., 2021).

Calcium ion channels play an essential role in chronic pain by participating in pain signal transmission and modulation. In chronic pain states, abnormal calcium channel activity can lead to hyperalgesia and central sensitization. For example, Cav2.2, also known as the N-type voltage-gated calcium channel, is widely present in the presynaptic membrane of nerve cells and plays a key role in regulating neurotransmitter release. The Cav2.2 (N-type) channel is a clinically relevant pain target, validated through genetic and pharmacological studies. CBD3063, a selective, first-in-class, CRMP2-based peptide mimetic, allosterically modulates Cav2.2 to achieve analgesia without negative side effects (Gomez et al., 2023).

The activation of glutamate receptors is closely related to central sensitization. The AMPA receptor, an ionotropic glutamate receptor, plays a crucial role in synaptic transmission and plasticity within the CNS. In chronic pain, abnormal AMPA receptor activity is associated with hyperalgesia and the development of central sensitization, particularly in the spinal dorsal horn. The enhanced activity of AMPA receptors is considered one of the core mechanisms of chronic pain (Lee et al., 2012; Li et al., 2014). Excessive activation of spinal glutamate NMDARs is also a critical mechanism in chronic neuropathic pain. Research has shown that both presynaptic and postsynaptic NMDARs in the spinal cord play distinct roles in regulating nociceptive input from different types of neuronal injury, with NMDARs in primary afferent excitatory neurons maintaining chronic pain induced by chemotherapy and nerve injury (Huang et al., 2023). The transition from acute to chronic pain and the subsequent maintenance of pain are complex physiological processes, with NMDA receptors (NMDARs) being a key factor and a primary target for emerging pain therapies such as ketamine (Yang Y. et al., 2020).

Other related receptors also play vital roles in pain transmission. For example, TRPV1 is a non-selective cation channel that exists in the somatosensory system, particularly in primary afferent neurons that respond to harmful or potentially harmful stimuli (nociceptors). Stimulation of TRPV1 induces a burning sensation, reflecting its core role in pain. Modern TRPV1-targeted drugs may rapidly emerge as the next generation of analgesics for a wide range of pain conditions (Iftinca et al., 2021). Capsaicin, for instance, selectively activates TRPV1, which is abundant in nociceptive primary afferent neurons, and this mechanism forms the basis for capsaicin-induced burning pain (Arora et al., 2021). Additionally, GPR151 is highly expressed in dorsal root ganglia and is closely linked to pain regulation, particularly in neuropathic pain. GPR151 modulates the function of P2X3 receptors and activates microglia, playing a role in pain processing (Xia et al., 2021).

### 3.2 Gene and epigenetics

Building upon the pathological dysregulation at molecular and cellular levels, epigenetic regulation emerges as a central interface bridging environmental stimuli and transcriptional reprogramming. This biological orchestrator facilitates the conversion of transient nociceptive inputs into enduring genomic imprints through DNA methylation, histone modification, and related epigenetic processes, thereby offering a multiscale framework to investigate the maladaptive transition from acute pain to chronic persistence (Mauceri, 2022).

#### 3.2.1 Gene variations and chronic pain

Significant genomic linkages exist between polymorphic gene profiles and persistent pain susceptibility. A prime exemplar involves the SCN9A gene product Nav1.7-a voltage-sensitive sodium channel subunit serving as a molecular linchpin in acute-to-chronic pain conversion pathways, as previously discussed. Pathogenic variants in this locus underlie SCN9A-associated neuropathic spectrum disorders (SCN9A-NSD), a rare monogenic condition characterized by neuropathic pain phenotypes comprising three clinical entities: inherited erythromelalgia (IEM, an autosomal-dominant genodermatosis), paroxysmal severe pain disorder (PSPD), and sodium channel-associated small fiber neuropathy (SCN9A-SFN) (Drenth and Waxman, 2007; Caldito et al., 2024).

Deciphering the molecular architecture of nociception-associated genetic variants and their allelic diversity yields promising therapeutic targets for pharmacological interventions that modulate transcriptional activity or functional protein cascades, enabling precision medicine approaches to pain mitigation. Empirical investigations demonstrate clinically relevant correlations between specific single-nucleotide polymorphisms-particularly PAR2 (rs2243057) and IL-17A (rs3819025) gene variants-and nociceptive hypersensitivity or immune-dysregulation phenotypes. Specifically, these genomic markers exhibit statistically robust associations with enhanced vulnerability to persistent pain states across diverse patient cohorts (Soeda et al., 2023).

#### 3.2.2 Epigenetic modifications in pain transition

DNA methylation and demethylation are fundamental epigenetic processes that significantly influence the progression of pain, especially in the transformation from acute to chronic pain states (Mauceri, 2022; Descalzi et al., 2015). Recent research has investigated how these epigenetic modifications control genes involved in nociception and the perception of pain, as well as the evolving patterns of these mechanisms during the development of chronic pain (Xiong et al., 2024). These dynamic alterations are orchestrated by DNA methyltransferases (DNMT) and ten-eleven translocation (TET) enzymes, which modulate the activity of genes associated with both pro-nociceptive and anti-nociceptive pathways. The article further explores how DNA methylation reprogramming contributes to the progression from acute to chronic pain, highlighting the critical need to unravel the temporal patterns of DNA methylation changes during this transition. Such insights are essential for advancing the development of precise therapeutic strategies aimed at addressing chronic pain.

For example, chronic pain caused by nerve injury is associated with persistent DNA methylation reprogramming in the dorsal root ganglion (DRG). Using methylation-sensitive restriction enzyme analysis, one study quantitatively measured whole-genome DNA methylation changes and found that spinal nerve ligation induced changes at 8% of CpG sites in the DRG, with hypomethylation being especially prevalent outside CpG islands in regions such as introns, intergenic areas, and repetitive sequences (Garriga et al., 2018). These epigenetic changes were sustained across both acute and chronic phases of pain, and reducing DNA methylation was shown to exacerbate pain sensitivity, while restoring methylation alleviated symptoms. This indicates that DNA methylation remodeling in the DRG is a key factor in the development and persistence of neuropathic pain. Reduced DNA methylation was found to induce hyperalgesia, while increased DNA methylation alleviated neuropathic pain (Garriga et al., 2018). Further research has also examined the effects of peripheral nerve injury on DNA methylation at various time points (1 day, 2 weeks, 6 months, and 1 year) in mice. Using a spared nerve injury model, the study conducted an epigenome-wide analysis of the mouse PFC, finding that spared nerve injury caused rapid and persistent DNA methylation changes, with significant methylation differences observed at all time points between the injured and sham-operated mice (Topham et al., 2020).

**Figure 2:**
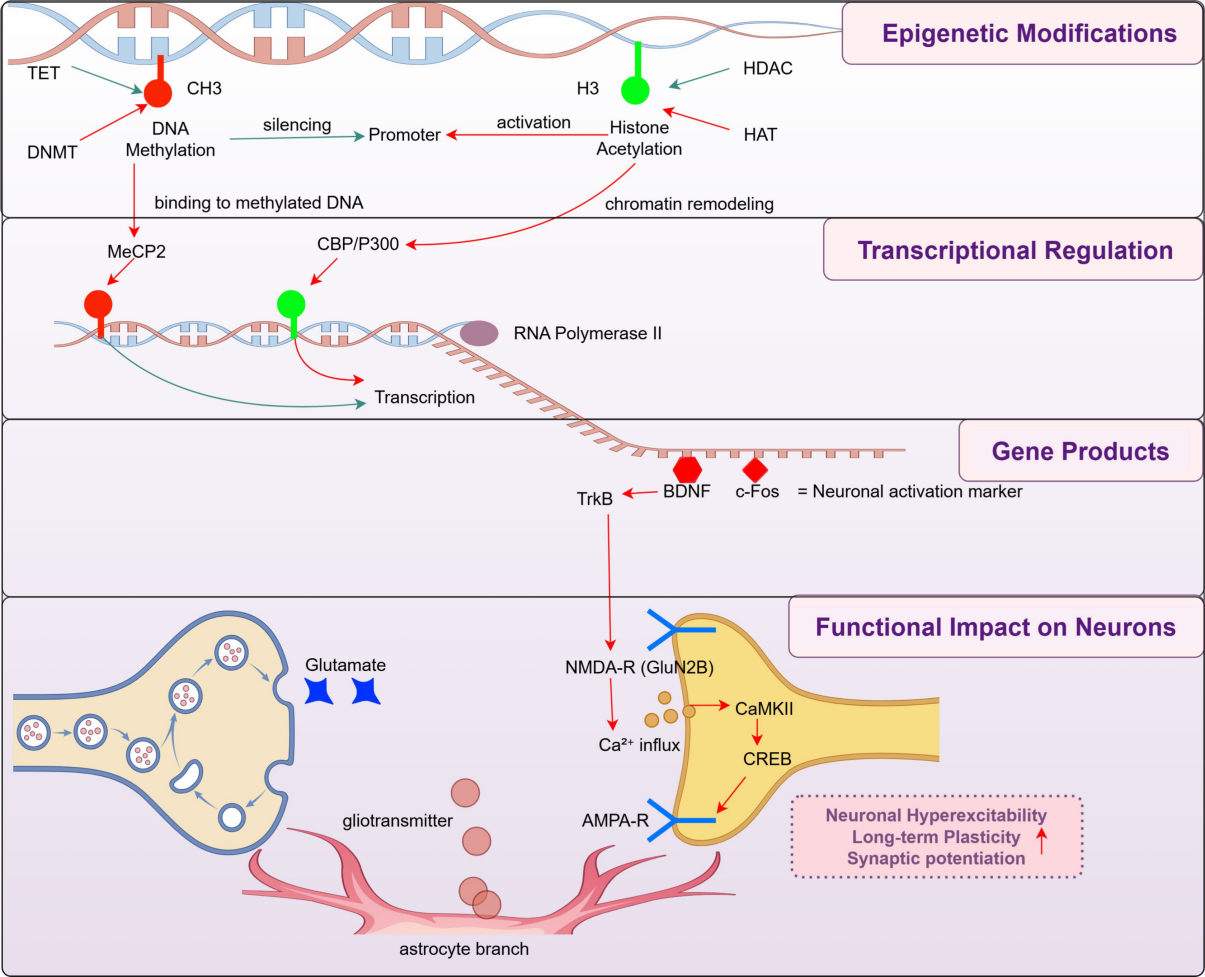
Epigenetic regulation of synaptic plasticity in pain chronification. This schematic representation of how epigenetic modifications (top), including DNA methylation and histone acetylation, influence gene transcription and downstream neuronal function in the context of chronic pain. This mechanistic cascade-spanning from chromatin remodeling to gene transcription, neurotrophic signaling, and synaptic modulation-is organized across four hierarchical modules: epigenetic modifications, transcriptional regulation, gene products, and functional impact on neurons. These modules collectively illustrate how early epigenetic cues can propagate through molecular cascades to induce neuronal hyperexcitability, long-term plasticity, and central sensitization. This framework highlights a mechanistic rather than time-based definition of pain chronification and incorporates both bottom-up molecular signaling and top-down modulation via activity-regulated transcription factors such as CREB.

Histone modifications also play a crucial role in gene expression and pain regulation. Studies have shown that histone modifications are essential in neurodevelopment and neurological diseases. Histone methylation and acetylation have been implicated in neuropathic pain, with these modifications influencing chronic pain through inflammatory mediators (Park et al., 2022; Jiang et al., 2022). In particular, histone acetylation is regulated by histone acetyltransferases (HATs), which promote gene transcription, and histone deacetylases (HDACs), which repress gene expression. Dysregulation of the HDAC/HAT balance has been associated with aberrant expression of pain-related genes and central sensitization, highlighting their therapeutic relevance. These epigenetic modifications ultimately influence gene transcription and neuronal function. For instance, BDNF upregulation under epigenetic control can potentiate NMDA receptor-mediated calcium influx and trigger the activation of the CaMKII-CREB signaling cascade, reinforcing excitatory synaptic transmission. In parallel, AMPA receptor trafficking and phosphorylation also contribute to synaptic potentiation in pain-relevant circuits. Importantly, immediate early genes such as c-Fos are often upregulated in spinal and supraspinal neurons in response to persistent nociceptive input and are widely used as markers of neuronal activation in chronic pain models. Beyond neuronal signaling, excessive glutamate release and astrocytic gliotransmitter signaling can further potentiate excitatory synapses and contribute to central sensitization. Collectively, these changes lead to long-term synaptic plasticity, promoting heightened pain sensitivity and persistence (Figure 2).

### 3.3 In the nervous system: structure and function

A growing body of evidence highlights the significant role of inflammation in both the peripheral and central nervous systems (PNS and CNS) in the development of chronic pain. The impact of inflammation, whether occurring early or late, can differ substantially, functioning as either a protective mechanism or a contributing factor to pain progression. For instance, damage caused by pain triggers the activation of glial and immune cells in the PNS, which release pro-inflammatory molecules that sensitize nociceptors, ultimately leading to chronic pain. Similarly, within the CNS, neuroinflammation drives central sensitization, further facilitating the transition to persistent pain. Conversely, macrophages and glial cells in both the PNS and CNS can also play a beneficial role by releasing anti-inflammatory molecules and specialized pro-resolving mediators (SPMs), which actively contribute to the resolution of pain (Fang et al., 2023; Chen et al., 2023). This dual role of inflammation underscores its complex and multifaceted influence on pain dynamics.

#### 3.3.1 Transformations in the central nervous system

The formation of chronic pain is accompanied by structural changes in the brain, such as changes in gray and white matter volumes. For example, in trigeminal neuralgia patients, gray matter volume decreases in areas such as the anterior cingulate gyrus and superior/middle frontal gyrus. Compared with healthy controls, trigeminal neuralgia patients show reductions in gray matter volume in several brain regions, including the bilateral middle temporal gyrus, bilateral superior/middle frontal gyrus, left precentral gyrus, right fusiform gyrus, and anterior cingulate gyrus. Voxel-based morphometry studies have found reductions in gray matter volume in regions such as temporal-pole-sup-R and precentral-R, with gray matter volume in these areas correlated with disease duration, pressure point cross-sectional area, and quality of life scores in trigeminal neuralgia patients. The anterior cingulate cortex, a key structure in the limbic system, is involved in regulating cognitive control, reinforcement-based learning, pain, emotion, and motor functions (Ge et al., 2023).

Studies have found that chronic pain patients exhibit increased functional connectivity in the default mode network (DMN), particularly with the posterior cingulate cortex/precuneus (PCC/PCu) and posterior cingulate cortex (Kucyi et al., 2014; Čeko et al., 2020). This suggests that chronic pain may be related to enhanced functional connectivity in certain brain regions that are typically active at rest and suppressed during task performance. Functional connectivity between the mPFC and nucleus accumbens (NAc) predicts the transition from acute to chronic pain states. In patients with subacute back pain, increased functional connectivity between the mPFC and NAc is associated with sustained pain perception, suggesting that changes in the cortical-striatal circuit are related to the transition from acute to chronic pain (Baliki et al., 2012; Löffler et al., 2022).

Electroencephalogram (EEG) studies on the transition from acute to chronic pain show several key electrical activity changes: during pain stimulation, the most common EEG change is an increase in delta activity (mainly in the contralateral frontal area). Results regarding theta activity during pain stimulation are inconsistent, with some studies reporting an overall increase, while others show a decrease or no significant change. The most commonly reported EEG change is a reduction in alpha activity (mainly in the parietal-occipital region), although some studies report an increase or no significant change. Almost all studies report an increase in beta activity during pain stimulation, particularly in the temporal regions. Most studies show an increase in gamma activity during pain stimulation, observed in the frontal, central, and temporal regions. Delta coherence increases during pain stimulation between electrodes T5 in the temporal region and electrodes in the frontal, central, and parietal regions of the left hemisphere (contralateral to stimulation). Additionally, strong temporal coherence is observed from the left posterior region to the right anterior region and central region.

#### 3.3.2 Changes in the peripheral nervous system

The initial alteration observed in the peripheral nervous system is sensitization, which can be categorized into two main types: the sensitization of nociceptors and modifications at the nerve terminals. Nociceptor sensitization operates through both direct and indirect mechanisms. For example, the TRPV1 ion channel can be directly triggered by a rise in temperature or by chemical signals released from various sources, including resident cells like mast cells, recruited immune cells such as polymorphonuclear leukocytes (PMNL), as well as epithelial cells, Schwann cells, fibroblasts, and postganglionic sympathetic neurons (SPGN). This multifaceted process underscores the complexity of nociceptor activation and its role in peripheral sensitization. Once nociceptors are activated, the involved second messenger pathways interact at multiple levels, including calcium ions (Ca^2+^), effector molecules like protein kinase Cε (PKCε), and common targets such as TRPV1 and Nav1.8 (Pinho-Ribeiro et al., 2017). Neurotrophic factors play a critical role in nociceptor sensitization and hyperalgesia after tissue damage. These factors include the NGF family (NGF, brain-derived neurotrophic factor, neurotrophin-3, and neurotrophin-4) and the glial cell-derived neurotrophic factor family (glial cell-derived neurotrophic factor, neurturin, artemin, and persephin). The receptors for these neurotrophic factors are used to classify primary nociceptors and non-nociceptors. Since these factors are essential in maintaining the afferent phenotype, including neurotransmitter expression and the composition of specific ion channels and transducers, their loss or increase can significantly enhance the excitability of nociceptors (Sikandar et al., 2018; Merighi, 2024).

Peripheral tissue inflammation or nerve damage can cause significant changes in the distribution of peripheral nerve afferent terminals in the spinal dorsal horn. Normally, different types of afferent fibers terminate in different laminae of the spinal dorsal horn, transmitting various sensory signals. However, under chronic pain conditions, the distribution of these terminals may change. Structural changes in the neural tissue of the spinal dorsal horn include alterations in the number of dendritic spines in neurons. After sensory nerve damage, the axon diameter and neuronal cell body size significantly decrease, and some neurons may even die, leading to a reduction in the density of pain receptors in the epidermis. After peripheral nerve damage, sympathetic nerve axon terminals sprout and invade the surrounding sensory neurons, forming a so-called “basket-like structure” (Kuner and Flor, 2016).

Peripheral nerve damage also affects the CNS (spinal cord), and these changes, in turn, influence the regeneration of damaged nerves. For instance, apoptosis of spinal anterior horn cells and synaptic stripping after peripheral nerve damage reduces the speed of nerve regeneration (Chu et al., 2022). Pathological changes in the spinal dorsal horn contribute to sensory abnormalities after peripheral nerve injury, such as ectopic discharges in the dorsal root ganglion that enhance pain signal transmission (Jang and Garraway, 2024). After peripheral nerve damage, axonal sprouting occurs at the proximal injury site, reinnervating the skin and muscles. However, due to the slow regeneration rate of axons, nerve regeneration is delayed.

In spinal cord injury regeneration and repair studies, spinal cord injury regeneration is described as one of the most challenging medical problems, suggesting a link between regenerative impairment after nerve injury and chronic pain. In pain management, the use of biomaterials and regenerative medicine is actively being explored, with progress made in improving the efficacy of conventional drug therapies and as a novel non-pharmacological approach for chronic pain caused by degenerative diseases.

### 3.4 Psychosocial mechanisms and the transition to chronic pain

Psychosocial influences are increasingly recognized as pivotal modulators in the transition from acute to chronic pain. These factors do not act independently but interact dynamically with neuroplastic remodeling and nociceptive signaling, contributing to maladaptive feedback loops that reinforce central sensitization and hinder pain resolution (Song et al., 2024). Rather than being merely peripheral phenomena, chronic pain is now understood as a result of structural and functional brain changes, particularly within circuits associated with emotion, motivation, and cognition (Urien and Wang, 2019). Therefore, although the previous sections have already discussed the molecular and cellular mechanisms, epigenetic regulation, and neuroplasticity changes involved in pain chronification, converging evidence underscores the critical role of cognitive-affective brain networks-particularly corticolimbic and prefrontal-limbic circuits-in modulating pain perception, emotional reactivity, and behavioral adaptation. Functional and structural changes in the prefrontal cortex, amygdala, hippocampus, and nucleus accumbens have been shown to predict the transition to chronic pain and mediate its persistence (Vachon-Presseau et al., 2016; Taylor, 2018; Thompson and Neugebauer, 2019). Importantly, these circuits are capable of both amplifying and mitigating pain experiences, depending on the interplay between risk and protective psychosocial factors.

#### 3.4.1 Neurocognitive modulation and individual vulnerability

Cognitive and personality traits-including age, attentional capacity, learning history, and baseline resilience-can shape individual pain trajectories by influencing pain perception, coping strategies, and neuroadaptive responses. Adaptive cognitive strategies such as attentional reappraisal and positive expectancy can dampen nociceptive processing, whereas maladaptive patterns-especially pain catastrophizing-are strongly associated with exaggerated threat perception, heightened pain intensity, and delayed recovery. Clinical data suggest that individuals who exhibit elevated levels of catastrophizing before surgery are more likely to develop chronic postoperative pain, a trajectory linked to deficient endogenous analgesic systems and altered cortical appraisal pathways (Sambo et al., 2010).

#### 3.4.2 Emotional dysregulation and affective circuits

Affective states modulate pain through bidirectional pathways involving the limbic system and its descending projections. Positive affect and emotional resilience may enhance prefrontal inhibitory control over thalamocortical and brainstem circuits, thereby attenuating pain signals (Hanssen et al., 2013). In contrast, sustained negative affect-including fear, anxiety, and depressive symptoms-potentiates nociceptive transmission via amygdala-mediated noradrenergic facilitation, promoting spinal sensitization and central amplification (Bushnell et al., 2013). Experimental paradigms have shown that emotionally charged visual stimuli alter pain perception: negatively valenced images intensify pain responses, whereas positively valenced stimuli exert analgesic effects. These findings underscore the importance of affective context in shaping both acute pain experiences and long-term chronification risk.

#### 3.4.3 Behavioral patterns and pain maintenance

Behavioral responses to pain-such as avoidance, persistence, or engagement-are mediated by complex neurocognitive mechanisms and can significantly affect the trajectory of pain chronification. Avoidance behaviors, particularly those reinforced by fear learning, are linked to increased disability, reduced functional recovery, and amplified cortical pain representation. Pain catastrophizing represents a prototypical maladaptive behavioral response with distinctive neurofunctional signatures, including heightened activity in the medial prefrontal cortex and limbic areas (Janke et al., 2016). Conversely, interventions promoting behavioral activation and re-engagement-such as graded exposure or operant-based therapy-have shown efficacy in disrupting these maladaptive cycles and restoring function (Louw et al., 2024).

**Figure 3:**
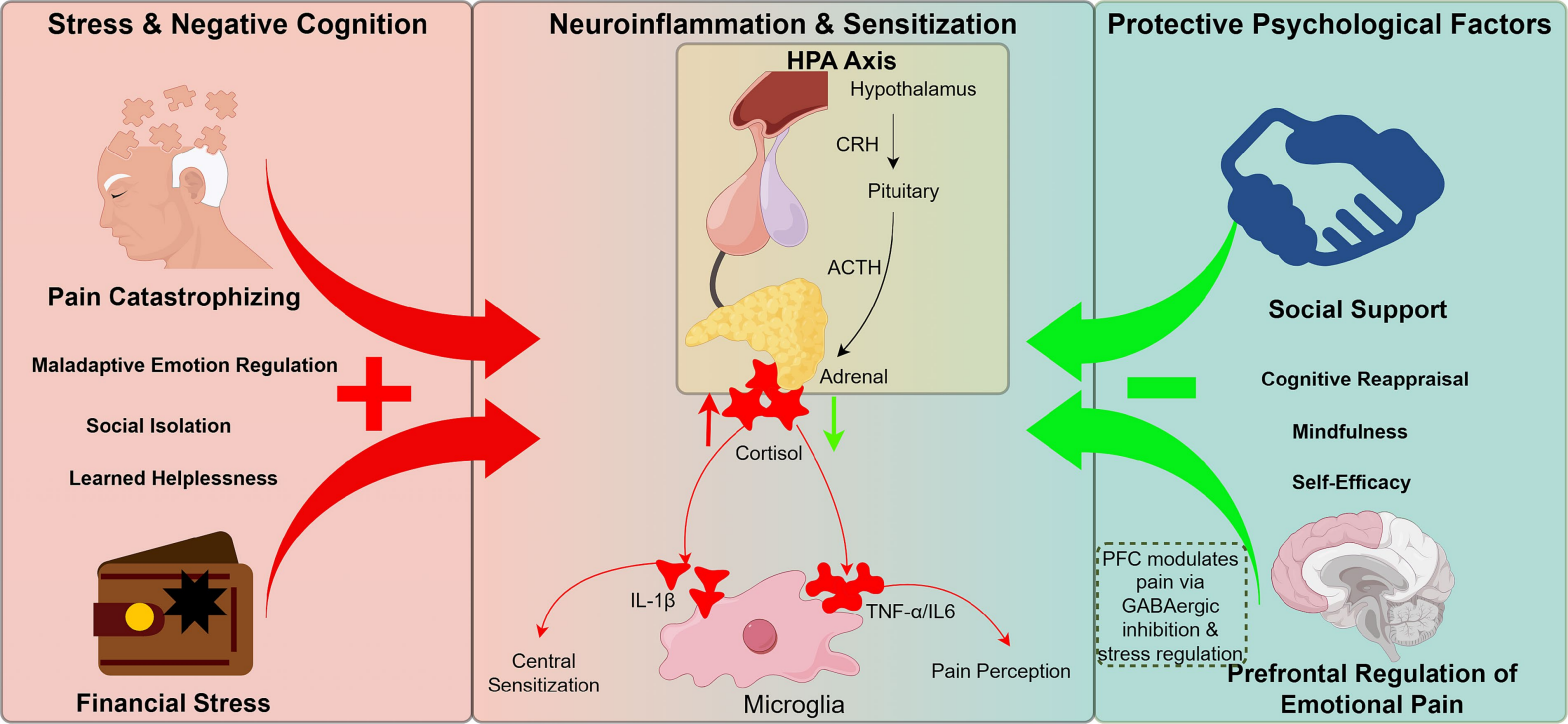
Bidirectional influence of psychosocial factors on pain chronification through neuroendocrine feedback circuits. Negative modulators (e.g., pain catastrophizing, financial stress, social isolation) activate the HPA axis, elevating cortisol and pro-inflammatory cytokines (e.g., IL-1β), which sensitize microglia and promote central sensitization. Chronic stress disrupts PFC regulation of limbic circuits and impairs GABAergic inhibition. Conversely, positive psychosocial factors (e.g., social support, emotional resilience, cognitive reappraisal) buffer HPA hyperactivation and restore top-down PFC control, exerting anti-nociceptive effects. This framework emphasizes the bidirectional role of psychosocial states in shaping chronic pain trajectories.

Together, these findings highlight the bidirectional and modulatory nature of psychosocial mechanisms in pain chronification. While certain traits and emotional patterns increase vulnerability, adaptive cognitive, emotional, and behavioral strategies may provide resilience and support pain resolution (Louw et al., 2024; Edwards et al., 2016). This balance offers a compelling rationale for integrated interventions that target not only risk factors but also bolster protective mechanisms. Neuroendocrine and immune pathways further mediate the influence of psychosocial context on pain chronification. Chronic stress has been shown to hyperactivate the HPA axis, resulting in elevated cortisol and proinflammatory cytokines such as IL-1β, TNF-α, and IL-6, which sensitize microglia and exacerbate central sensitization (Aboushaar and Serrano, 2024). Simultaneously, stress disrupts the top-down inhibitory control exerted by the PFC over limbic circuits and impairs GABAergic signaling, contributing to heightened emotional and affective dimensions of pain (Edwards et al., 2016). In contrast, positive psychosocial resources-including social support, emotional resilience, and cognitive reappraisal-can attenuate HPA axis overactivation and enhance PFC-driven regulatory control, ultimately engaging stress-buffering and anti-nociceptive mechanisms. These converging pathways highlight the dual potential of psychosocial influences to either aggravate or mitigate chronic pain development, depending on their directionality and neurobiological impact. Psychosocial factors, such as stress and social support, exert bidirectional control over pain chronicity through neuroendocrine pathways, as shown in Figure 3.

## 4 Conclusion and outlook

The transition from acute to chronic pain is a complex, evolving process that involves the interplay of multiple factors, including molecular, cellular, genetic, epigenetic, neuroplastic, and psychosocial elements. This review explores the key mechanisms driving this transition: ongoing activation of inflammatory mediators and immune responses, which alter pain signaling through both peripheral and central sensitization; glial cells (such as microglia and astrocytes), which contribute to synaptic plasticity and neuroinflammation in the central nervous system; functional disruptions in ion channels (such as Nav1.7, Cav2.2) and receptors (including NMDA and TRPV1), which exacerbate neuronal hyperexcitability; genetic variations (like those in SCN9A and COMT) and epigenetic changes (such as DNA methylation and histone modifications), which influence pain-related gene expression and establish a genetic vulnerability to chronic pain. Additionally, psychosocial factors, including pain catastrophizing, anxiety, and depression, enhance pain perception through neuroendocrine pathways and corticostriatal circuits, contributing to a reinforcing cycle.

Current research continues to face significant challenges, primarily because most mechanistic studies focus on isolated components rather than the dynamic interactions among multiple physiological systems, which are essential for understanding the complexity of chronic pain disorders (Giordano, 2008). Furthermore, clinical translation is impeded by bottlenecks (Denk and McMahon, 2017), such as limited efficacy of targeted therapies like Nav1.7 inhibitors due to off-target effects and inadequate blood-brain barrier penetration. The absence of early predictive biomarkers also hampers timely diagnosis and intervention (Amirdelfan et al., 2020).

Beyond identifying mechanistic domains, this review also emphasizes the importance of temporally stratified intervention strategies. As detailed in Sections 3.1–3.4, the transition from acute nociception to chronic pain progresses through distinct phases-acute, transition, and chronic-each marked by specific biological features (see Figure 1). These phases are defined here based on the dominant cellular and molecular processes described in preclinical and translational studies, rather than on rigid clinical timeframes. This evolving pathophysiology creates critical windows for mechanism-informed intervention. In the acute phase, inhibitors of early inflammatory cascades-such as anti-NGF agents or IL-1β blockers-along with glial-modulatory therapies like TLR4/MyD88 pathway antagonists or cathepsin S inhibitors, may help attenuate the neuroimmune interactions that drive peripheral and central sensitization (Chi, 2016; Dai et al., 2020; Yang H. et al., 2020). In the transition phase, when epigenetic remodeling becomes prominent-for example, BDNF silencing in the dorsal root ganglion (DRG) and H3K27me3-mediated gene repression-epigenetic therapies such as DNMT inhibitors and HDAC modulators may help reverse maladaptive transcriptional changes (Song et al., 2011; Silva-Llanes et al., 2023). In the chronic phase, neuromodulatory approaches (e.g., spinal cord stimulation, transcranial magnetic stimulation) combined with behavioral and cognitive rehabilitation may help restore top-down control and circuit balance (Noga and Guest, 2021; Toledo et al., 2021; Liampas et al., 2020). These time-specific interventions align directly with the four mechanistic domains outlined in this review-molecular-cellular inflammation, epigenetic dysregulation, neuroplastic remodeling, and psychosocial amplification-thus providing a mechanism-informed and temporally guided framework for future therapeutic development.

Building on this temporally guided framework, we further propose a comprehensive future-oriented roadmap comprising four strategic directions: mechanism-based precision, objective pain evaluation, treatment innovation, and cognitive-emotional integration. This translational roadmap spans from basic research to clinical intervention, aiming to improve early identification, accurate assessment, targeted treatment, and long-term recovery in chronic pain care. Notably, among these pillars, the domain of treatment innovation offers preliminary mechanistic alignment with the four key drivers of pain chronicization described in this review-including molecular-cellular inflammation, epigenetic dysregulation, maladaptive plasticity, and psychosocial amplification. While still conceptual, these alignments underscore the potential of tailoring future therapies to specific pathophysiological layers of chronic pain, thereby promoting more effective, personalized interventions. Future research should prioritize integrating multi-omics and neuroimaging technologies in longitudinal studies to identify dynamic biomarkers of the transition from acute to chronic pain and to detect high-risk populations (Petre et al., 2022; Lee et al., 2022). Equally critical is the development of refined pain assessment tools-such as wearable devices, multiplex biomarker panels, and real-time digital tracking platforms-that can capture nuanced fluctuations in pain, improving both clinical decision-making and research fidelity (Leroux et al., 2021; Avila et al., 2021). Precision-targeted therapies should also be explored, including highly selective drugs for Nav1.7, Cav2.2, and other ion channels, using nanodelivery systems to optimize central nervous system targeting. Additionally, epigenetic tools like CRISPR-dCas9 could be employed to modulate aberrant methylation or histone changes in pain-related genes (Denk and McMahon, 2017). Interdisciplinary approaches combining neuromodulation techniques (e.g., transcranial magnetic stimulation, spinal cord stimulation) with cognitive-behavioral therapy [e.g., Cognitive behavioral therapy (CBT)] offer promise for disrupting pain-emotion feedback loops by modulating corticostriatal connectivity (Knotkova et al., 2021; Urits et al., 2019). Lastly, AI-driven models leveraging large clinical datasets may improve risk stratification and support personalized early interventions, such as preventive anti-inflammatory treatments or targeted psychosocial care (Sajdeya and Narouze, 2024).

**Figure 4:**
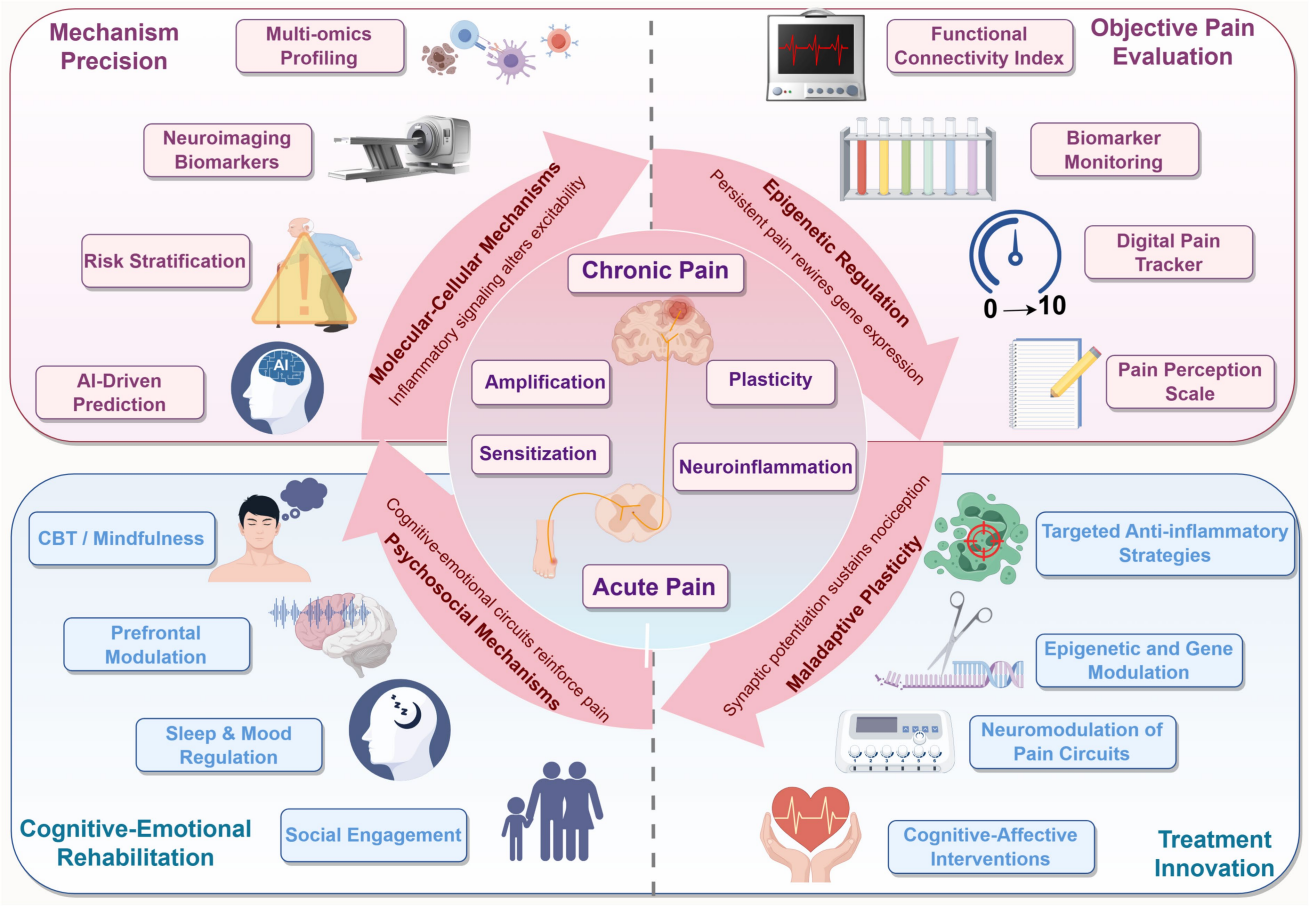
Integrated framework of chronic pain mechanisms and future translational strategies. The central illustration depicts the progression from acute to chronic pain, driven by four interconnected domains: molecular-cellular inflammation, epigenetic reprogramming, neuroplastic remodeling, and psychosocial feedback. Each domain forms a layer in the maladaptive cycle of pain. Surrounding segments outline future directions including biomarker-based risk stratification, multi-dimensional pain assessment, targeted and combinatorial therapies, and cognitive-emotional rehabilitation. This schematic bridges mechanistic understanding with clinical innovation, offering a roadmap toward precision pain prevention.

Ultimately, a unified framework that connects early identification, continuous evaluation, mechanism-based intervention, and emotional-cognitive rehabilitation will be essential for reshaping chronic pain care. As depicted in Figure 4, this approach moves the field beyond symptom suppression, toward truly personalized and mechanism-driven prevention.
